# Combining liver stiffness with hyaluronic acid provides superior prognostic performance in chronic hepatitis C

**DOI:** 10.1371/journal.pone.0212036

**Published:** 2019-02-11

**Authors:** Janne Fuglsang Hansen, Karen Mølgaard Christiansen, Benjamin Staugaard, Belinda Klemmensen Moessner, Søren Lillevang, Aleksander Krag, Peer Brehm Christensen

**Affiliations:** 1 Department of Infectious Diseases, Odense University Hospital, Odense, Denmark; 2 Department of Clinical Research, Faculty of Health Sciences, University of Southern Denmark, Odense, Denmark; 3 Clinical Immunological Department, Odense University Hospital, Odense, Denmark; 4 Department of Gastroenterology and Hepatology, Odense University Hospital, Odense, Denmark; Medizinische Fakultat der RWTH Aachen, GERMANY

## Abstract

**Background:**

Non-invasive methods are the first choice for liver fibrosis evaluation in chronic liver diseases, but few studies investigate the ability of combined methods to predict outcomes.

**Methods:**

591 chronic hepatitis C patients with baseline liver stiffness (LSM) by FibroScan and hyaluronic acid measurements were identified retrospectively. The patients were grouped by baseline LSM: < 10kPa, 10–16.9kPa, and 17-75kPa. Primary outcomes were all-cause mortality and liver-related mortality, analyzed using cox regression and competing risk regression models, respectively.

**Results:**

Median follow-up was 46.1 months. Prevalence of cirrhosis at baseline was 107/591 (18.1%). Median LSM was 6.8kPa (IQR 5.3–11.6) and divided into groups, 404/591 (68.4%) had a LSM < 10kPa, 100/591 (16.9%) had a LSM between 10–16.9kPa and 87/591 (14.7%) had a LSM between 17-75kPa. There were 69 deaths, 27 from liver-related disease. 26 patients developed cirrhosis and 30 developed complications of cirrhosis. The mortality rate in the 17-75kPa group was 9.7/100 person-years, compared to 2.2/100 person-years and 1.1/100 person-years in the 10–16.9kPa and <10kPa groups (p<0.005). Liver-related mortality increased 10-fold for each group (p<0.005). Cirrhotic complications occurred almost exclusively in the 17-75kPa group, with an incidence of 10.3/100 person-years, compared to 1.8/100 person-years and 0.2/100 person-years in the 10–16.9kPa and <10kPa groups (p<0.005). Median hyaluronic acid in the 17-75kPa group was approximately 200ng/mL. Patients with a LSM 17-75kPa had significantly higher risks of death, liver-related death, and complications to cirrhosis if their hyaluronic acid measurement was more than or equal to 200ng/mL at baseline, with hazard ratios of 3.25 (95% CI 1.48–7.25), 7.7 (95% CI 2.32–28), and 3.2 (95% CI 1.35–7.39), respectively.

**Conclusions:**

The combination of LSM and circulating hyaluronic acid measurements significantly improved prognostic ability, relative to LSM alone. Combined static and dynamic markers of liver fibrosis could provide superior risk prediction.

## Introduction

Chronic hepatitis C (CHC) is associated with the development of liver fibrosis, which can lead to cirrhosis after years of infection. Cirrhosis is associated with high liver-related morbidity and mortality, and curing CHC before severe fibrosis or cirrhosis develops reduces this risk [1]. Identifying patients at risk of fibrosis progression would improve prognosis prediction and enable the physician to choose the correct treatment and surveillance program for each patient.

Liver stiffness measurement (LSM) with vibration-controlled transient elastography technology has become standard practice for non-invasive liver fibrosis diagnosis and monitoring [2]. In 2011, Vergniol et al. found that only 66% of patients with baseline LSM (bLSM) >20kPa and 77% of patients with bLSM more than or equal to 9.5kPa were alive after five years, compared to 96% of patients with bLSM < 9.5kPa. Other studies have also shown the ability of bLSM to predict long-term outcomes, although bLSM cut-off values and outcome measures vary [3–11]. Vergniol et al. also showed that by adding the commercial test Fibrotest (FT) to LSM, they significantly increased the model’s performance for five year survival [3].

Furthermore, international recommendations state that fibrosis degree diagnosis should be measured by two different non-invasive tests, as concordance of more than one test increases the diagnostic ability and discordance requires further investigation [12, 13].

Hyaluronic acid (HA) is a major component of the connective tissues in the body and is produced in the liver by activated hepatic stellate cells as a part of the wound-healing response. During chronic liver inflammation, such as that occurring during CHC infection, there is continuous hepatic stellate cell activation and therefore increased HA synthesis. The liver is also the primary organ responsible for HA degradation. Both the increased synthesis and decreased clearance during fibrogenesis increases HA concentration [14–16], making HA a candidate for measuring liver fibrosis. Several studies have shown that HA measurement can rule-out cirrhosis with high negative predictive values >90%, but has less potential for diagnosing lesser degrees of fibrosis [16–19].

Baseline HA (bHA) is also able to predict mortality and complications, although reported cut-off values and outcome variables also vary [20–24]. The study with the longest follow-up assessed a human immunodeficiency virus (HIV) and hepatitis co-infected population for a median of 8.2 years and showed that the risk of hepatic encephalopathy or liver-related death increased 5-fold for patients with bHA >75ng/mL and 30-fold for patients with bHA >250ng/mL, compared to patients with normal bHA [21].

We hypothesized that a combination of baseline LSM and HA would improve the prognostic value of the individual test and the objective of the study was to investigate whether the prognostic value of bLSM could be improved when combined with bHA in patients with CHC.

## Patients and methods

The study was a retrospective study including all patients with CHC followed at a single university hospital from the implementation of FibroScan (Echosens, France) on May 1, 2007 until December 31, 2014. Baseline was defined as the date of their first reliable LSM. The LSM was done either with the medium or from 2009 with the XL probe (S1 and S2 Tables). Reliability criteria for LSM were more than 10 valid scans and an IQR/median ≤ 30% if the median stiffness >7.0kPa [25]. Patients with chronic hepatitis B (CHB), HIV, or liver transplant prior to baseline were excluded, and patients with no bHA measurement within 180 days of their bLSM were also excluded. There were no liver transplantations in the follow-up period. For overall mortality and liver related mortality observation ended on the day of death or on December 31, 2014. The observation period for complications ended on the date of the first complication, the date of absence from the clinic as determined by the clinician or on December 31, 2014 if the follow-up was ongoing. A history of alcohol consumption was defined as 30/20 gram/day for men/women in accordance with the National Danish cut-off values for high alcohol consumption [26]. Some of the data presented in this study were previously reported as part of a 5 year follow-up of CHB and CHC patients [27].

Patients were divided into the following three groups based on bLSM: bLSM<10kPa, bLSM between 10–16.9kPa, and bLSM 17-75kPa. The high cut-off was described by Degos et al. as the cut-off with highest likelihood gain information on cirrhosis with a probability of cirrhosis of 72% in a cohort with a 13.8% pre-test probability [28]. The lower cut-off was chosen based on the previous observation that patients with an bLSM <10kPa showed spontaneous decreases in repeated measurements indicating that at least part of the LSM was not explained by fibrosis and that the actual fibrosis degree in many cases were lower than the single measure indicated [27]. Therefore, the cut-offs were chosen as surrogate cut-offs for mild fibrosis (<10kPa), moderate/severe fibrosis (10–16.9kPa) and cirrhosis (17-75kPa). Patients were treated as recommended during their follow-up but was not included if they had SVR prior to first reliable LSM. None of the measurements of bLSM or bHA was performed during treatment. Serum HA was measured by ELISA (Corgenix, Co., USA). Measurement range was 10–800 ng/ml.

The primary outcomes were death from any cause or death from liver-related disease. Secondary outcome was complications of cirrhosis defined as the first incidence of either hepatocellular carcinoma (HCC), variceal bleeding, ascites or hepatic coma. Cirrhosis was determined by one of the following: biopsy with a METAVIR-score F4, decompensation, or a reliable bLSM equal to or more than 17kPa with no decrease to <17kPa, if a second scan was performed. Diagnosis of cirrhosis within 180 days of bLSM was classified as cirrhosis at inclusion.

Data was extracted from The Danish National Database for Hepatitis B and C (DANHEP) and a local quality control database (INFCARE). Data missing from the databases was supplemented with patient medical record reviews if necessary. LSMs were extracted from the FibroScan hard disk, and data was merged using the Danish national PIN (a 10-digit personal identification number).

This project was approved by The Danish Patient Safety Authority (j.nr 3-3013-2243/1) and the Danish Data Protection Agency (2006-41-7196 and 2012-41-0079).

### Statistical analysis

The significance level was set at a p-value < 0.05. Proportions and medians were compared using Pearsons Chi^2^ and Kruskal-Wallis median test. Nonparametric ROC analyses were used to generate sensitivity, specificity, negative predictive value (NPV), and positive predictive value (PPV) for death or liver-related death during follow-up. For creating receiver operating characteristic (ROC) curves dependent on both bLSM and bHA logistic regression was used with transformation of LSM and HA. Cox regression for all-cause death and competing risk regression for all other events were used to estimate the hazard ratio and subhazard ratio. Continuous variables were transformed in regression models if necessary. Sustained viral response was forced into all survival models. The effects of sex, age, a history of alcohol use or intravenous drug use (IDU) were tested along with LSM and HA, and variables found to be significant in univariate analysis were included in the multivariate models. The models were checked for significant interactions and reduced using backward elimination, comparing models with likelihood ratio tests and Akaike information criterion for competing risk regression models [29]. Assumptions and goodness of fit of the regression models were tested as appropriate. Cut-offs for HA was chosen as the median HA by baseline LSM group in order to achieve models with highest power. The cut-offs for optimal sensitivity and specificity, 90% sensitivity and specificity found by ROC analysis was tested as well. All analysis was performed with STATA 15.0.

## Results

**Fig 1:**
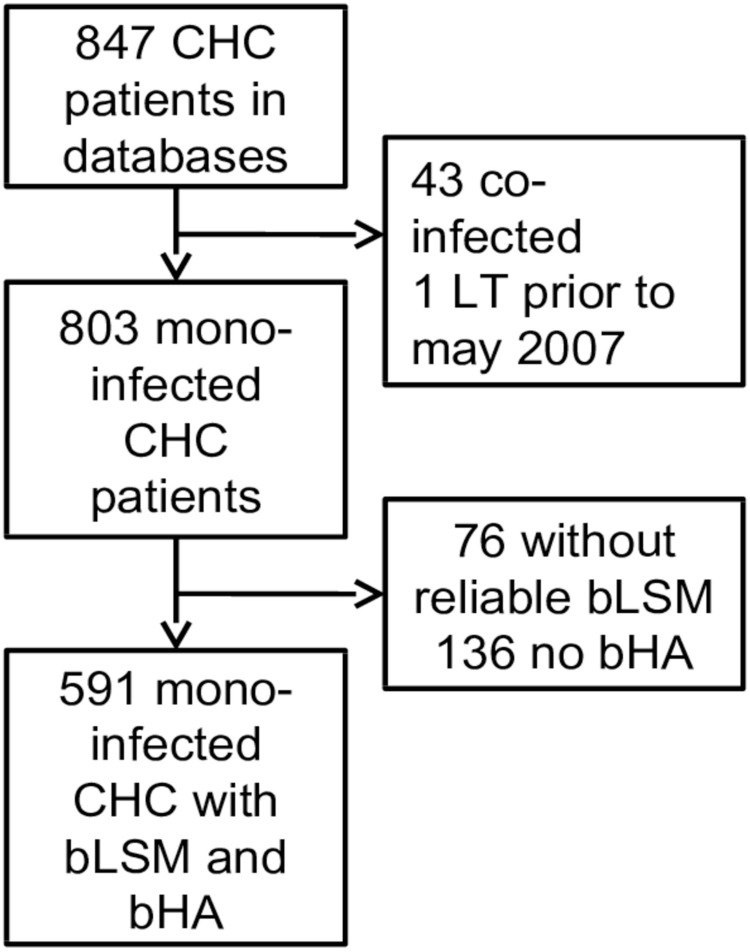
Flow chart for inclusion. LT; liver transplantation.

43 of the 847 patients with CHC evaluated for inclusion were co-infected with CHB/HIV, and 1 patient had a liver transplant (LT) prior to bLSM. 76 patients had no reliable LSM, and 136 had no blood samples at bLSM (11/136 also had SVR prior to first reliable LSM) (Fig 1). The excluded patients were younger (median 44.4 vs. 46.1 years of age, p = 0.040) and a larger proportion were male (75% vs. 64.1%, p = 0.006). Death was more frequent among the excluded patients, with 48 deaths (22%) compared to 68 deaths (11.5%) among the included patients (p = 0.001). There was no significant difference in deaths due to liver-related disease, with 14 (33.3%) and 27 (39%) liver-related deaths, respectively (p = 0.166). The baseline characteristics of the patients who died in the excluded group were not significantly different from the characteristics of the patients who died in the included group.

The final study population included 591 mono-infected CHC patients with a reliable bLSM and a bHA within 180 days. The median follow-up to either death or end of observation was 48.2months (IQR 23.6–67.3). Seventy patients achieved sustained viral response (SVR), and 12/70 of them were treated with direct-acting antivirals. The baseline characteristics are summarized in Table 1.

**Table 1:** Baseline characteristics of the entire cohort and divided into subgroups according to baseline LSM.

**Baseline characteristics**	**Overall** **(n = 591)**	**Baseline LSM <10kPa (n = 404)**	**Baseline LSM 10–16.9kPa (n = 100)**	**Baseline LSM 17-75kPa (n = 87)**	**p-value**
	n/median (IQR or %)	n/median (IQR or %)	n (IQR or %)	n (IQR or %)	
**Age**	46.1 (38.2–53.8)	43.5 (36.1–51.6)	49.8 (43.8–54)	53.6 (46.8–56.9)	0.0001
**Male sex**	379 (64.1)	247 (61.1)	66 (66)	66 (75.9)	0.031
**Western European**	508 (86)	345(85.4)	85 (85)	78(89.7)	0.558
**Genotype**					0.709
**(n = 362/93/77^a^)**					
**1**	244 (45.8)	165 (45.6)	43 (46.2)	36 (46.8)	
**2**	45 (8.5)	30 (8.3)	8 (8.6)	7 (9.1)	
**3**	233 (43.6)	160 (44.2)	40 (43)	32 (41.6)	
**4**	10 (1.9)	7 (1.9)	1 (1.1)	2 (2.6)	
6	1 (0.2)	0	1 (1.1)	0	
**Cirrhosis at baseline**	108 (18.3)	1 (0.25)	20 (20)	87 (100)	<0.005
**History of alcohol use**	324 (54.8)	215 (53.2)	55 (55)	54(62.1)	0.322
**History of IDU**	442 (74.8)	305 (75.5)	78 (78)	59 (67.8)	0.235
**HA in ng/mL**	36 (18–71)	27 (15–44)	62 (36–94)	196 (92–416)	0.0001
**ALT in U/L (342/75/76)**^a^	59 (38–105)	55 (36–93)	72 (45–135)	75 (46–145)	0.0006
**Platelet count in 10^9/L (336/75/75)**^a^	215 (168–262)	232 (193–273)	203 (158–266)	122 (81–172)	0.0001
**Albumin in g/L (342/76/76)**^a^	43 (40–45)	43 (40–46)	42 (40–44)	39 (35–43)	0.0001
**Bilirubin in** **(343/76/76)**^a^	8 (6–12)	8 (6–10)	9 (7–13.5)	12 (9–20.5)	0.0001
**INR** **(327/79/76)**^a^	1 (0.9–1.1)	1 (0.9–1)	1 (1–1.1)	1.1 (1–1.3)	0.0001

IDU; intravenous drugs, ALT; alanine aminotransferase.

^a^Number of patients with baseline sample in baseline LSM 10kPa/10-16.9kPa/17-75kPagroups.

### Mortality

Sixty-eight (11.5%) patients died during follow-up, and the all-cause mortality rate was 2.9/100 person-years (py) (IQR 2.3–3.6). The median follow-up for the patients who died was 28.2months (IQR18.2–41.7, range 4.1–90.5).There was no significant difference between the lower (6.4% (26/404) deaths, 1.1/100py) and middle (10% (10/100) deaths, 2.2/100py) bLSM groups (p = 0.453), but the mortality rate was significantly higher at 9.7/100py (36.8% (32/87) deaths) for patients with a bLSM17-75kPa(p <0.0005).

The 17kPa cut-off had a NPV for death of 92.9% during follow-up, and a bHA cut-off of 117ng/mL similarly had a NPV of 90% (Table 2). The risk of dying was independently associated with increasing age, bLSM, and bHA, and the risk of death associated with increasing bLSM and bHA values are included in the supplementary material (S1 and S2 Figs). The HR of both bLSM and bHA was still significant in a multivariate model adjusted for SVR (Table 3). A model adjusted for age is included in the supplementary material (S3 Table).

**Table 2:** Baseline LSM and HA for predicting death, liver-related death and first cirrhotic complication during follow-up. The tables shows the used cut-offs for LSM and HA as well as cut-offs yielding 90% sensitivity or specificity for each individual test.

		Cut-off	Patients above cut-off (%)			Sens (%)	Spe (%)	PPV (%)	NPV (%)
**Baseline LSM (kPa)**	**All-cause death**	AUC 0.70 (95% CI 0.62–0.78)							
	**LSM 10–16.9kPa** **LSM 17-75kPa**	10	31.6			61.8	72.3	22.5	93.6
		17	14.7			47.1	90.0	40.0	92.9
	Cut-off for 90% sensitivity	5	79.7			90.0	21.6	12.9	94.2
	Cut-off for 90% specificity	17	14.7			47.1	90.0	40.0	92.9
	**Liver-related death**^a^	AUC 0.93 (95% CI 0.89–0.98)							
	LSM 10–16.9kPa	10	31.6			96.3	71.5	14.0	99.8
	LSM 17-75kPa	17	14.7			85.2	88.7	26.7	99.2
	Cut-off for 90% sensitivity	14	20.1			90.0	83.2	20.5	99.4
	Cut-off for 90% specificity	19	13.0			85.2	90.0	29.1	99.2
	**First cirrhotic complication**^b^	AUC 0.89 (95% CI 0.82–0.97)							
	LSM 10–16.9kPa	10	28.1			90.0	75.4	17.0	99.3
	LSM 17-75kPa	17	11.2			66.7	91.9	31.5	98.0
	Cut-off for 90% sensitivity	12	20.4			90.0	83.5	22.8	99.3
	Cut-off for 90% specificity	15	12.8			73.3	90.0	29.0	98.4
**Baseline HA (ng/mL)**	**All-cause death**	AUC 0.68 (95% CI 0.60–0.76)							
	Median HA for group 17-75kPa	200	9.3			39.4	95.4	52.7	92.4
	Cut-off for 90% sensitivity	14	84.9			90.0	15.9	12.2	92.4
	Cut-off for 90% specificity	117	14.6			45.5	90.0	37.2	92.7
	**Liver-related death**^a^	AUC 0.93 (95% CI 0.88–0.99)							
	Median HA for group 17-75kPa	200	9.3			76.9	94.6	40.7	98.8
	Cut-off for 90% sensitivity	40	45.2			90.0	57.6	9.3	99.2
	Cut-off for 90% specificity	125	14.0			84.6	90.0	29.0	99.2
	**First cirrhotic complication**^b^	AUC 0.89 (95% CI 0.83–0.94)							
	Median HA for group 17-75kPa	200		6		46.7	93.3		28		96.9
	Cut-off for 90% sensitivity	62		26.3		90	73.8		16		99
	Cut-off for 90% specificity	131		10.1		70	90		28		98.2
**Combined model^c^**	**All-cause death**	AUC 0.74 (95% CI 0.67–0.80)^d^							
	**Liver-related death**^a^	AUC 0.94 (95% CI 0.91–0.98)^d^							
	**First cirrhotic complication**^b^	AUC 0.91 (95% CI 0.85–0.98)							

Sens; sensitivity. Spe; Specificity. PPV; positive predictive value. NPV; negative predictive value

^a^ Model for liver-related deaths for patients >30 years of age.

^b^ Model for cirrhotic complications excluding the patients <30 years and the 29 patients who already had a complication at baseline.

^c^Combined model for LSM and HA. Cut-offs are displayed in the supplementary material (S4 Table).

^d^Significantly higher AUC for death/death from liver disease than bLSM or bHA alone (p = 0.034 and 0.001/0.001 and 0.006, respectively)

**Table 3:** Significant predictors of death from all-cause, liver-related death or cirrhosis development corrected for achieving sustained viral response (SVR).

**Event**	**Predictors**		**Univariate**		**Multivariate**	
			**HR (95% CI)**	**p-value**	**HR (95% CI)**	**p-value**
**Death from all causes**	Baseline LSM					
		<10kPa	Reference		Reference	
		10–16.9kPa	1.33 (0.63–2.76)	0.446	1.09 (0.50–2.34)	0.835
		17-75kPa	6 (3.55–10)	<0.005	3.64 (1.65–7.9)	0.001
	Baseline lnHA		1.93 (1.59–2.36)	<0.005	1.37 (1.03–1.83)	0.031
	Baseline age		1.03 (1.01–1.06)	0.004	NS	-
	Male sex		1.66 (0.97–2.85)	0.064	NS	-
	Ever excessive alcohol use		1.6 (0.97–2.64)	0.063	NS	-
	Ever intravenous drug use		0.94 (0.55–1.62)	0.830	NS	-
	SVR		0.46 (0.19–1.15)	0.094	0.32 (1.03–1.83)	0.014
**Event**	**Predictors**		**Univariate**		**Multivariate**	
			**sHR (95% CI)**	**p-value**	**sHR (95% CI)**	**p-value**
**Liver-related death**^a^	Baseline LSM					
		<10kPa	Reference		Reference	
		10–16.9kPa	9.5 (0.98–91.3)	0.051	4.04 (0.41–39.8)	0.176
		17-75kPa	97 (13.2–713)	<0.005	11 (1.22–98.6)	0.018
	Baseline lnHA		5.2 (3.43–7.95)	<0.005	3.15 (1.77–5.62)	<0.005
	Baseline age		1.07 (1.03–1.11)	<0.005	NS	-
	Male sex		1.97 (0.79–4.87)	0.144	NS	-
	Ever excessive alcohol use		1.96 (0.86–4.49)	0.109	NS	-
	Ever intravenous drug use		2.1 (0.97–4.59)	0.060	NS	-
	SVR		1.09 (0.67–3.22)	0.883	0.69 (0.22–2.14)	0.523
**Complications**^b^	Baseline LSM					
		<10kPa	Reference		Reference	0.034
		10–16.9kPa	9.6 (2.5–36.6)	0.001	4.3 (1.12–18.3)	0.001
		17-75kPa	59 (17.4–200)	<0.005	9 (2.49–32.2)	
	Baseline lnHA		4.5 (3.4–5.9)	<0.005	2.8 (1.9–3.8)	<0.005
	Baseline age		1.07 (1.03–1.11)	<0.005	NS	
	Male sex		1.59 (0.70–3.59)	0.262	NS	
	Ever excessive alcohol use		0.8 (0.39–1.66)	0.556	NS	
	Ever intravenous drug use		1.83 (0.87–3.84)	0.113	NS	
	SVR		0.84 (0.25–2.83)	0.779	0.39 (0.1–1.35)	0.189

^a^Analysis of subpopulation of (560/591) patients with a baseline age above 30 years of age.

^b^ subpopulation (531/591) >30 years of age and no previous complications.

HR; Hazard ratio. lnHA; natural logarithm of bHA. NS; non-significant.

**Fig 2:**
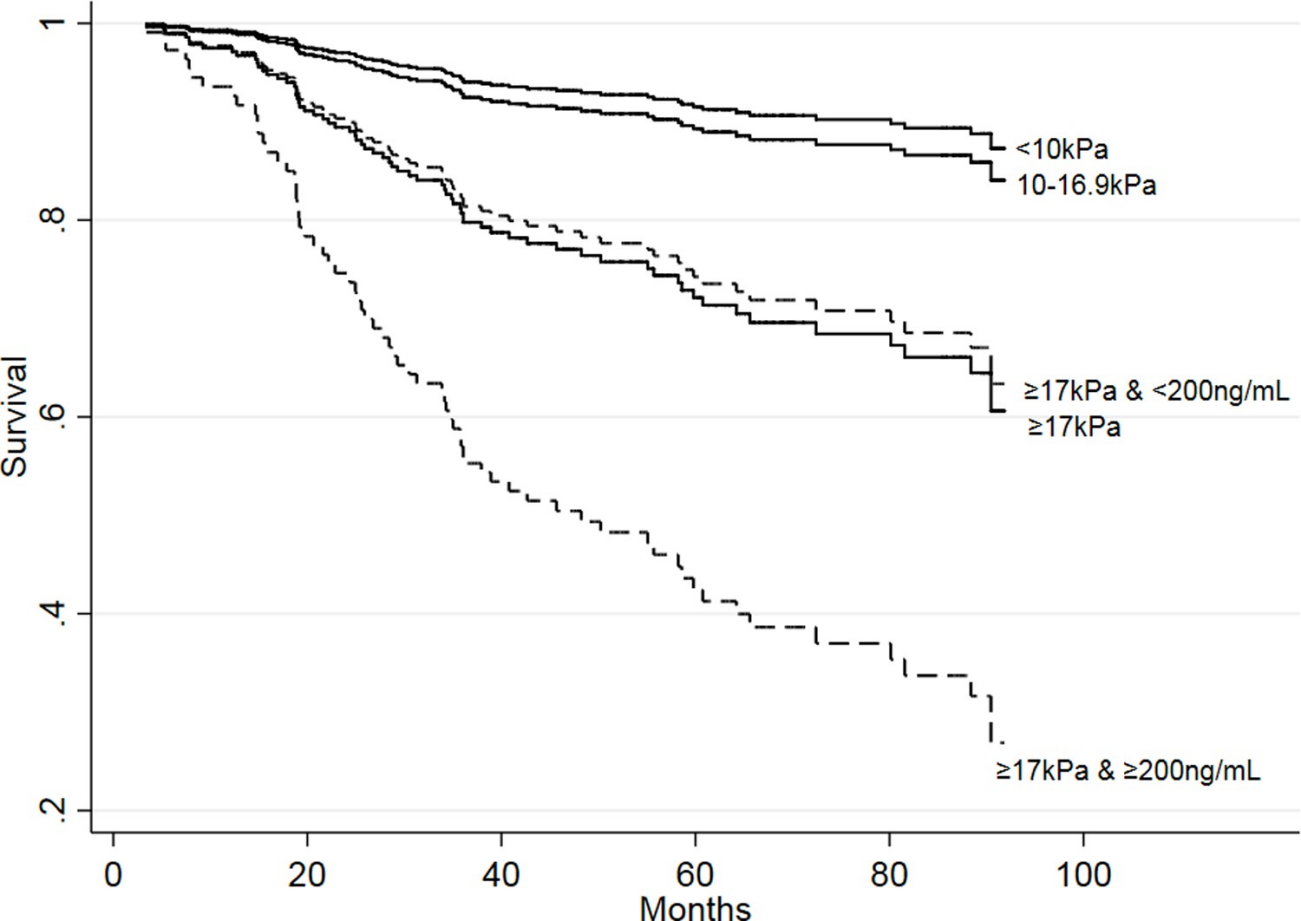
Overall survival stratified by baseline LSM groups. Within the baseline 17-75kPa group the survival is further stratified by baseline HA (dotted lines), p = 0.004.

The median bHA was 196ng/mL among patients with bLSM 17-75kPa. The addition of an approximation of the median value of 200ng/mL to the 17kPa cut-off further increased the predictive power, as patients with a bHA more than or equal to 200ng/mL had a 3.25 (95% CI 1.48–7.25) times higher risk of dying during follow-up than patients with a bHA < 200ng/mL (Fig 2). The model combining bLSM group and bHA >200ng/mL for predicting death during follow-up had an AUC of 0.74 (95% CI 0.67–0.80), and performed significantly better than the AUC for bLSM or bHA alone, p = 0.034 and p = 0.001, respectively. The bHA cut-off for 90% sensitivity and specificity in the group of patients with bLSM 17-75kPa was 100ng/mL and 484ng/mL, respectively. Both the low and high cut-off had NPV and PPV with relatively low accuracy (NPV of 85% and 71% and PPV 44% and 69%, respectively). The 200ng/mL cut-off had sensitivity, specificity, PPV and NPV for death in the bLSM 17-75kPa group of 69%, 62%, 51% and 77%, respectively. The optimized cut-off for bHA in the bLSM 17-75kPa group was 172 and the sensitivity, specificity, PPV and NPV was 81%, 60%, 54% and 84% (S3 Fig).

### Liver-related mortality

There was one death (0.2%) related to liver disease in the bLSM < 10kPa group, three (3%) in the bLSM 10–16.9kPa group, and 23 (26.4%) in the bLSM 17-75kPa group. The liver-related mortality increased 10-fold, with mortality rates of 0.07/100py, 0.7/100py, and 7.1/100py for the three groups respectively (p = 0.005) in accordance with the number of deaths. The median follow-up among the 27 patients was 31.3months (IQR 14.9–59.8). No patient with a baseline age under 30 years died from liver-related causes, regardless of bLSM and bHA (the range for the patients < 30 years of age was 3.3–10.2kPa and 5-67ng/mL, respectively). The subhazard ratio (sHR) associated with bHA and bLSM for liver-related death for patients over 30 years of age (560/591) are shown in Table 3. A model including all 591 patients and the interaction with age is included in the supplementary material (S5 Table). A cut-off of 17kPa yielded a NPV of 99.2% for dying from liver-related cause during follow-up (Table 2), and liver-related mortality also increased with increasing bHA and bLSM (trends p < 0.005) (S1 and S2 Figs). No patient with a bHA below 29ng/mL or bLSM below 6kPa died from liver-related disease (p < 0.0005) and log(bHA) was still associated with the risk of liver-related death with a sHR of 3.8 (95% CI 0.5–9.4) when excluding the baseline cirrhotic patients.

**Fig 3:**
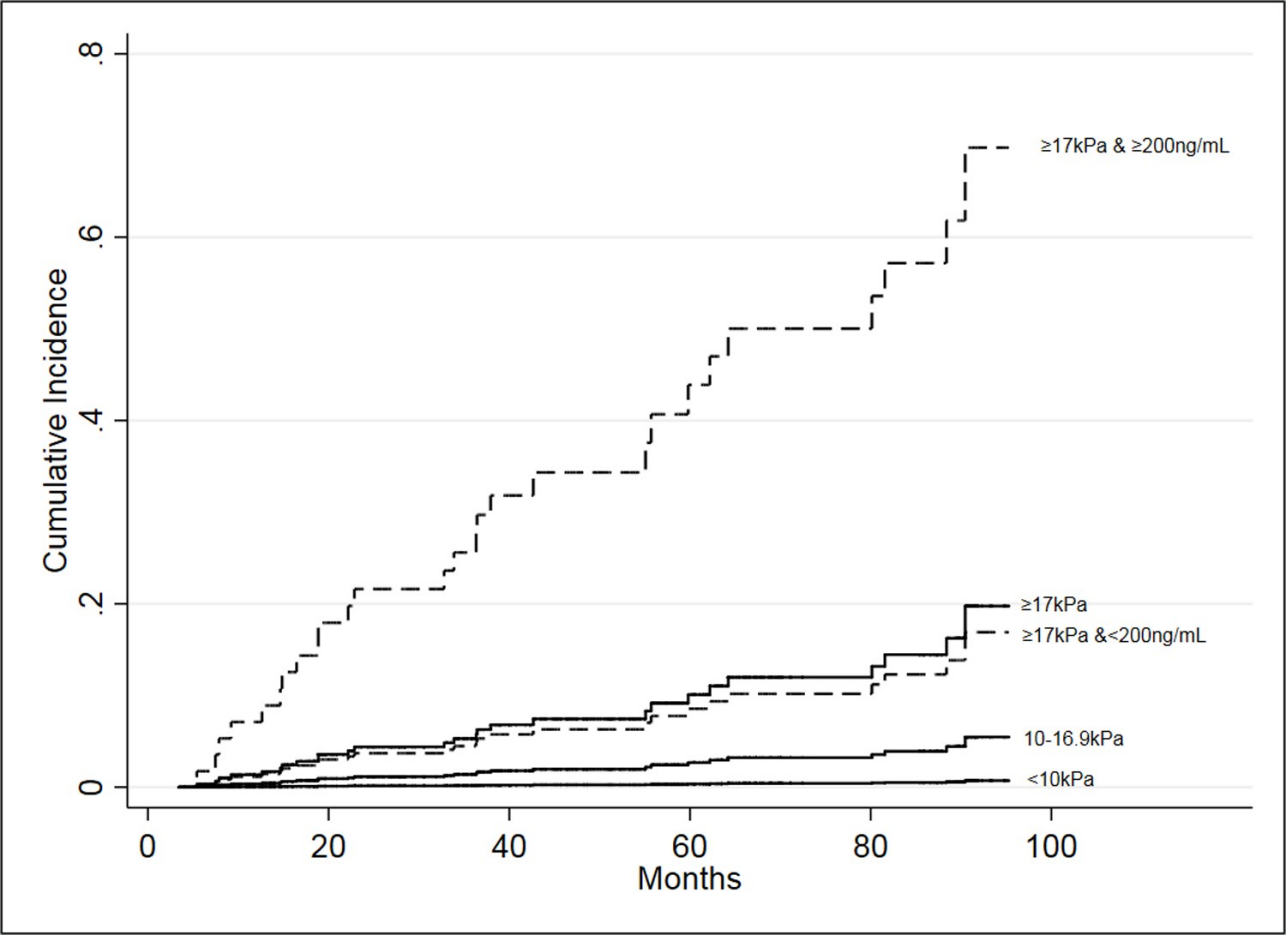
Cumulative incidence of liver-related deaths stratified by baseline LSM groups (solid lines). The group with bLSM 17-75kPa was further stratified by a bHA 200ng/mL cut-off (dotted lines).

Liver-related mortality could be further assessed by adding bHA to the high-risk bLSM 17-75kPa group, as the risk was 7.7 (95% CI 3.1–18.7, p 0.0005) times higher for patients with bHA of 200ng/mL or more (Fig 3). The sHR was not significant for death free of liver-death, neither by bLSM alone nor in the bLSM 17-75kPa group when stratified by bHA (S4 Fig and S6 Table). The model combining bHA equal to 200ng/mL or more and bLSM groups had an AUC for predicting liver-related death during follow-up of 0.94 (95% CI 0.91–0.98), and performed significantly better than both bLSM and bHA alone, p = 0.001 and p = 0.006, respectively. The 200ng/mL cut-off had sensitivity, specificity, PPV and NPV for the 17-75kPa group of 87%, 64%, 46% and 93%, respectively. The optimized cut-off for predicting liver related death among the patients with bLSM 17-75kPa group was 284ng/mL and had PPV and NPV of 57% and 88%. The 90% specificity cut-off for the entire cohort of 125ng/mL had sensitivity, specificity, PPV and NPV of 91, 42, 35%, 93% in the bLSM 17-75kPa group. Among the patients with bLSM <17kPa there were only 4 liver-related deaths, two from HCC and two from liver failure. For the three patients with bLSM 10–16.9kPa, none had a bHA less than the median (61.5ng/mL). The patient dying from liver related causes with a bLSM<10kPa had a bHA of 38ng/mL and a follow-up of 55.7months.

### Development of cirrhotic complications

The baseline prevalence of cirrhosis was 18.1% (107/591). 34 (31.8%) had a biopsy with cirrhosis, 10 (9.4%) was diagnosed by decompensation, 7 (6.5%) by variceal bleeding and 56 (52.3%) by LSM more than 16.9kPa. Baseline cirrhosis was associated with male sex (75% vs. 61.7%, p = 0.011) and older age (53.2 years, IQR 46.6–56.7 vs. 45.1 years, IQR 37.1–52.2, p = 0.001). The median bHA and bLSM for patients with cirrhosis was 165ng/mL (IQR 82–361) and 25.7kPa (IQR 17.3–45), compared to 29ng/mL (IQR 16–51) and 6.3kPa (IQR 5.1–8.4) among non-cirrhotic patients at baseline (both p = 0.0001).

Twenty-nine patients had a history of one or more complications at baseline and four of these within a month of their first reliable LSM (1 variceal bleeding, 1 HCC, 1 ascites and variceal bleeding and 1 patient who presented with all three) (S7 Table). Of the remaining patients, 5.3% (30/561) had a first episode of cirrhotic complications during the follow-up. Only 5/30 patients did not have cirrhosis at baseline. Cirrhosis was diagnosed by 17 (57%) LSM 17-75kPa, 1 (3%) variceal bleeding, 1 (3%) ascites, 11 (37%) biopsies. The incidence of cirrhotic complications increased with bLSM from 0.2/100py (0.7%, 3/404 patients) to 1.8/100py (7.4%, 7/95patients) and 10.3/100py (31.8%, 20/63 patients) in the <10kPa, 10–16.9kPa, and 17-75kPa groups, respectively (p < 0.005). Median follow-up for the 30 patients was 19.4 (IQR 5.6–32.1). The risk also significantly increased with increasing bHA (Table 3). The bHA cut-off for no complications during follow-up within the bLSM 17-75kPa group was 77ng/mL (19.5% of patients with bLSM 17-75kPa), p = 0.002 and had a sensitivity, specificity, PPV and NPV in the bLSM 17-75kPa group of 86.7, 81.5, 58.2 and 95.4, respectively. A bHA equal to or more than 200ng/mL increased the risk of complications 3.2 (95% CI 1.35–7.39) times for patients with a bLSM 17-75kPa (p = 0.006). The optimized cut-off for complications among the patients with bLSM 17-75kPa (228ng/mL) showed a similar risk of 4.4 (1.7–10.8, p = 0.001)(S5 Fig) and the risk of death without complications was not associated with LSM and HA (S6 Fig). Excluding the patients with baseline cirrhosis, log(bHA) still had a sHR of 2.7 (95% CI 1.2–5.5) for later cirrhotic complications in univariate analysis (p = 0.015) (S8 Table). Of the 30 patients with complications, 12 (40%) later died from liver related events. The median time from first complication to death from liver disease was 33months (IQR 3.1–48.9). Only 10 of the patients who had a first cirrhotic event during follow-up had a bLSM<17kPa and the median bHA for these 10 patients was 98ng/mL (IQR 43–167) versus 30ng/mL (IQR 16–52) among the rest of the patients with bLSM<17kPa (p = 0.002). The AUC for cirrhotic complications among the patients with bLSM<17kPa was 0.83 (95% CI 0.71–0.96). The optimized cut-off was 76.5ng/mL and produced sensitivity, specificity, PPV and NPV of 87, 81, 7 and 99, respectively. The univariate sHR was 11.4 (95% CI 3.3–39.2), p <0.0005)

## Discussion

We found that the risk of death was minimal for CHC patients with bLSM below 17kPa in this 7-year follow-up, and liver-related deaths occurred almost exclusively in the bLSM 17-75kPa group. Adding a bHA 200ng/mL cut-off to the LSM 17-75kPa group improved risk assessment for both all-cause and liver-related death significantly, allowing for further stratification of these high-risk patients. Complications of cirrhosis were also highly associated with bLSM 17-75kPa, but adding the bHA cut-off likewise improved the predictive power.

The low mortality seen in the bLSM <17kPa group is consistent with previous studies indicating that a high bLSM is associated with a higher risk of death, liver-related death, and liver-related events [3–9, 11, 30–32]. Our study also confirms previous studies showing that bHA is predictive of prognosis [20–24].

We did not find a significantly higher liver-related mortality for patients with bLSMs of 10–16.9kPa compared to patients with bLSM<10kPa. This was unexpected and may be due to the small number of events seen in our study or the length of the follow-up. However, the negative predictive value was very high when looking at the group with bLSM 17-75kPa, indicating that during a follow-up period of a median 48months, the patients are at an extremely low risk of dying due to their liver disease if their baseline LSM is below 17kPa. The risk of death was significantly higher for patients with bLSM >10kPa using a dichotomous cut-off of 10kPa which is in line with the 9.5kPa cut-off found to be associated with higher risk of death in the 2011 study by Vergniol et. al. However, our results indicate that this was due to the high risk for patients with LSM 17-75kPa which also identified patients likely to develop cirrhotic complications. Therefore, although the 17kPa cut-off is not a validated and optimized cut-off for cirrhosis, it may be useful to predict poor outcome in patients with advanced liver disease. As LSM<17kPa was an good predictor of liver-related death with a high NPV, bHA was used to identify high- and low risk groups within this group and the median used still had high NPV but increased the PPV substantially, identifying a “very-high risk” group, while the patients with bHA <200ng/mL showed the same risk as when identified by LSM more than or equal to 17kPa alone. Although the optimized cut-off in the group was higher (284ng/mL) and the PPV was higher for bHA>284ng/mL, the NPV for this cut-off was only 88%. As not missing patients at risk should be a high priority, the cut-off for the median was used, but which cut-off to use depends on how many high-risk patients it is acceptable to overlook in order to increase the PPV or how many it is acceptable to classify as at high risk to increase the NPV. LSM could also rule-out a risk of developing a cirrhotic complication with high NPV but as for liver-related death, the identification of patients likely to experience a complication was less accurate with the highest PPV for the 17kPa cut-off of 31%. Again bHA could increase the accuracy of the identification and by using the 200ng/mL cut-off, identifying a high-risk group among the patients with bLSM 17-75kPa.

Due to the low number of events, bHA was not added to a prediction model for liver related death excluding the group of patients with bLSM<17kPa. However, among the patients with bLSM <17kPa, the 10 patients who had a first cirrhotic complication during follow-up, had significantly higher bHA and a stratification by the optimal cut-off did show a significant difference in risk, leading us to believe that given a little longer follow-up, the association between a cut-off for bHA and liver-related death among the patients with low bLSM could also be shown. It was surprising that SVR had no protective association with death from liver disease or the development of cirrhotic complications, but this was probably due to the low number of patients achieving SVR in the period, as most patients were waiting for DAA treatment to become more generally available and the associations with the events were still significant when excluding the patients who achieved SVR (S9–S11 Tables).

None of the cut-offs could exclude death from any cause with NPV >90%. This is to be expected as death from non-liver and non-fibrotic disease should not display as high an association to LSM and HA as liver related disease and the association with death due to other causes than liver disease was also not significant in competing risk regression. However, as liver-related disease does comprise 39.7% of all deaths in this study, the association to overall mortality is still significant. As most of the deaths and complications occurred among the baseline cirrhotic patients, multivariate analysis of the non-cirrhotic group of patient was not possible, but this also indicated that either death or complications were unlikely to occur during the median 48months of follow-up, if the patient did not have cirrhosis at baseline.

The bHA cut-off used for stratifying the baseline 17kPa group can be debated but there is no real consensus on a HA value were the risk of liver-related death or complications is high. Two studies conducted among HIV co-infected showed that the risk of non-AIDS death was significantly higher if bHA above 75ng/mL, and patients with a bHA of more than 250ng/mL had a markedly high risk of hepatic encephalopathy during a 8year follow-up [21, 22]. However, the cohort is not directly comparable to ours as they had HIV and in some cases also CHB, which are both associated with increased risk of severe disease [33] and they did not combine bHA with other non-invasive tools. Most diagnostic studies reporting cut-offs for rule-in cirrhosis in the range 110-240ng/mL [16–19], and the 200ng/mL cut-off is probably also a second indicator of cirrhosis along with the 17kPa cut-off. Since HA is one of the oldest non-invasive markers, it would have been interesting to compare this with other widely available markers, such as FIB-4 or to adjust LSM for AST as it have been shown that lower AST values reduces the cut-offs for both advanced fibrosis and cirrhosis and LSM progression is less likely with AST<65U/L, indicating that it could be beneficial for prediction to correct for an inflammation marker [34–37]. However, routine liver tests in Denmark did not include aspartate-amino-transferases (AST) at the time of this study, therefore composite scores and adjustment based on AST were not possible. However, as the clinical outcomes are highly correlated with high LSM and high HA regardless of the reason for the high values and identifying high risk groups by LSM and HA is still reasonable, using the measurements as an indicator of prognosis rather than fibrosis degree.

There are few studies investigating combinations of widely recognized non-invasive tools for outcome prediction. Vergniol et al. combined LSM and FT and found an AUC of 0.907 for survival, an improvement relative to either LSM or FT alone [3]. This finding was confirmed in a large study of three French cohorts by Poynard et al. in 2014 [30] and Klibansky et al. also showed that a model including bLSM, MELD score, and AST/ALT ratio had superior predictive value for a composite event of death, decompensation, variceal bleeding, HCC, increase in Child Pugh score and listing for transplant during a two year follow-up [11]. Although these results are not directly comparable to this study, they are in agreement with our finding of better risk estimation when non-invasive measurements are combined. These, and our study, all combine LSM with some form of measure of either inflammation or fibrogenesis and the better prognostic value may be due to better identification of patients with active progressive disease.

The major limitation of our study is the relatively small number of patients, especially patients with events. This limitation probably also prevented us from using bHA and bLSM in combination among the non-cirrhotic patient to predict their outcome.

An additional limitation is the sizable percentage of our patients (25%) who could not be included in the study due to failure to keep their appointments for LSM or blood tests. Although there was no difference in the available baseline characteristics of the patients who died during the follow-up period and the excluded patients who died, there was an overall higher mortality among the excluded patients. The difference in overall mortality may be due to unmeasured characteristics, such as other co-morbidities or an ongoing drug use, since active intravenous drug users are at a higher risk for death by overdose or accident[38]. However, liver-related mortality was not significantly different among the excluded patients.

In conclusion, we found that our ability to predict the risk of death, liver-related death, and development of cirrhosis among patients with CHC was significantly improved if we combined baseline LSM with baseline HA measurements. Using two non-invasive tools in combination should be considered when making risk assessments, especially for patients with high LSM. This information may be especially useful when deciding on the intensity of disease surveillance, as well as for estimating the urgency of treatment in settings where treatment is not readily available to all. It may also help indicate which patients would benefit the most from anti-fibrotic agents in the future.

## Supporting information

S1 Fig Overall survival and liver-death free survival by different cut-offs for bLSM and bHA. Showing the overall survival by LSM (A) and HA (B) and liver-related death free survival by LSM (C) and HA (D).
(JPG)

S2 Fig **Cumulative incidence for liver related death by different cut-offs for LSM (A) and HA (B)**.
(JPG)

S3 Fig Overall survival stratified by bLSM and bHA. Overall survival by baseline LSM (solid lines) and the LSM 17-75kPa group stratified by the optimized baseline HA cut-off (dotted lines).
(JPG)

S4 Fig Liver-death free survival and cumulative incidence of liver-deaths and non-liver deaths stratified by bSLM and bHA. Liver-death free survival (A) by cox regression and (B) cumulative incidence for liver related deaths. (C) Shows the cumulative incidence of death with liver-related deaths as competing risk. All are stratified by LSM (solid lines) and in the LSM 17-75kPa group, the patients are further stratified by baseline HA (dotted lines).
(PNG)

S5 Fig Cumulative incidence of first complication stratified by bLSM and bHA. Baseline LSM are the solid lines and the group with baseline LSM 17-75kPa stratified by the optimized cut-off for baseline HA (dotted lines).
(JPG)

S6 Fig Cox regression and competing risk regression for complications and death without complications. The complication-free survival (A) using cox regression for the event of developing complications to cirrhosis and (B) the cumulative incidence of complications with death as competing risk. (C) shows the cumulative incidence of death with complications as the competing event. All are stratified by LSM (solid lines) and in the LSM 17-75kPa group, the patients are further stratified by baseline HA (dotted lines).
(JPG)

S7 Fig Scatterplot showing the baseline HA values by the baseline LSM values. The two vertical lines represents the 10kPa and the 17kPa cut-offs.
(JPG)

S1 Table Number of patients scanned with the median and XL probe and the median measure (IQR). P-value for difference in median.
(DOCX)

S2 Table Association for using XL probe with the risk of death, liver-related death and complications. The association with LSM and an interaction between probe and LSM was tested and none were significant. Medium probe is the reference. * Subhazard ratios with death from non-liver related causes as competing risk among the +30 years old.
(DOCX)

S3 Table Cox regression model for all-cause mortality with age in the multivariate model. (DOCX)

S4 Table Cut-offs for combined models for predicting outcome. Cut-offs for 90% sensitivity and 90% specificity for combined logistic models for all-cause death, liver related death and first cirrhotic complication ^a^ n = 560 (no patients <30years of age). ^b^ n = 531 (no patients <30years of age or with complications prior to or at inclusion).
(DOCX)

S5 Table Subhazard ratios for death from liver disease in for all patients. Competing risk regression with subhazard ratio for liver related death for all patients (n = 591) including the interaction between age and lnHA.
(DOCX)

S6 Table Competing risk regression for the event of non-liver related death. Death from liver-related causes is the competing risk.
(DOCX)

S7 Table Patients with complication prior to baseline. Baseline LSM and the time from the last complication to bLSM for the 29 patients with complications prior to or at the time of first reliable LSM and the complications registered.
(DOCX)

S8 Table The assocication of ln(Hyaluronic acid) with events among the non-cirrhotic population. * Subhazard ratios with death from non-liver related causes as competing risk among the +30 years old.** subhazard ratio for the patients >30years of age and without prior complications.
(DOCX)

S9 Table Association with death for the non-SVR patients. (DOCX)

S10 Table Association with death from liver disease among non-SVR patients. Competing risk regression for the non-SVR patients +30years of age (n = 493).
(DOCX)

S11 Table Association with complications among the non-SVR patients. Patients +30years of age, no previous complications (n = 493).
(DOCX)

S12 Table Mean time to events. (DOCX)

S13 Table Association of the baseline HA and LSM with overall death excluding the 10 patients who had a prior decompensation episode. (DOCX)

S14 Table Association of the baseline HA and LSM with liver-related death excluding the 10 patients who had a prior decompensation episode. (DOCX)
