# Supplementary figures and images for: Combining liver stiffness with hyaluronic acid provides superior prognostic performance in chronic hepatitis C
Source: PLoS One. 2019 Feb 11;14(2):e0212036. doi: 10.1371/journal.pone.0212036 (PMC6370278; doi:10.1371/journal.pone.0212036)

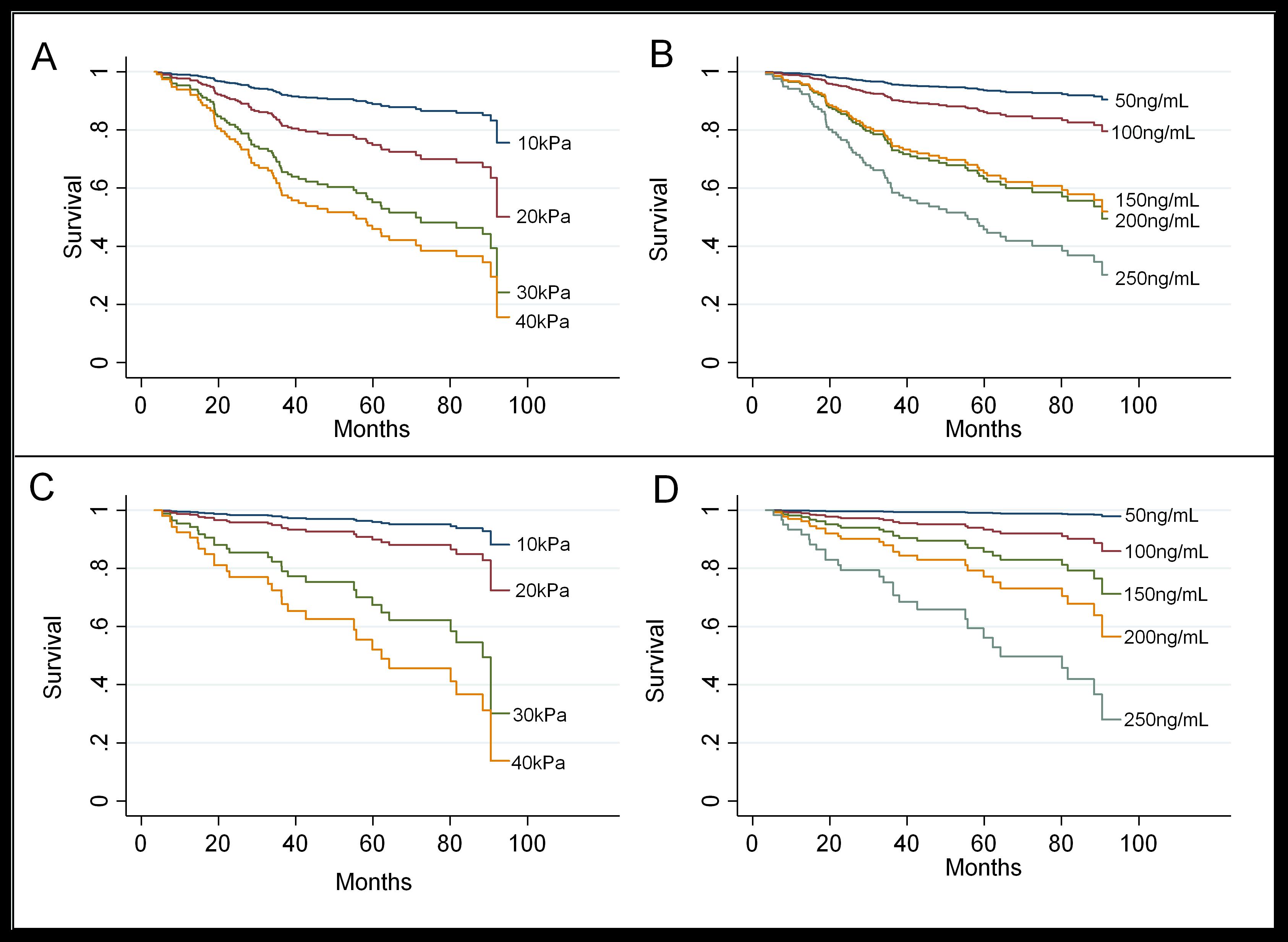

Supplement: S1 Fig — Showing the overall survival by LSM (A) and HA (B) and liver-related death free survival by LSM (C) and HA (D). (JPG) [file pone.0212036.s001.jpg]

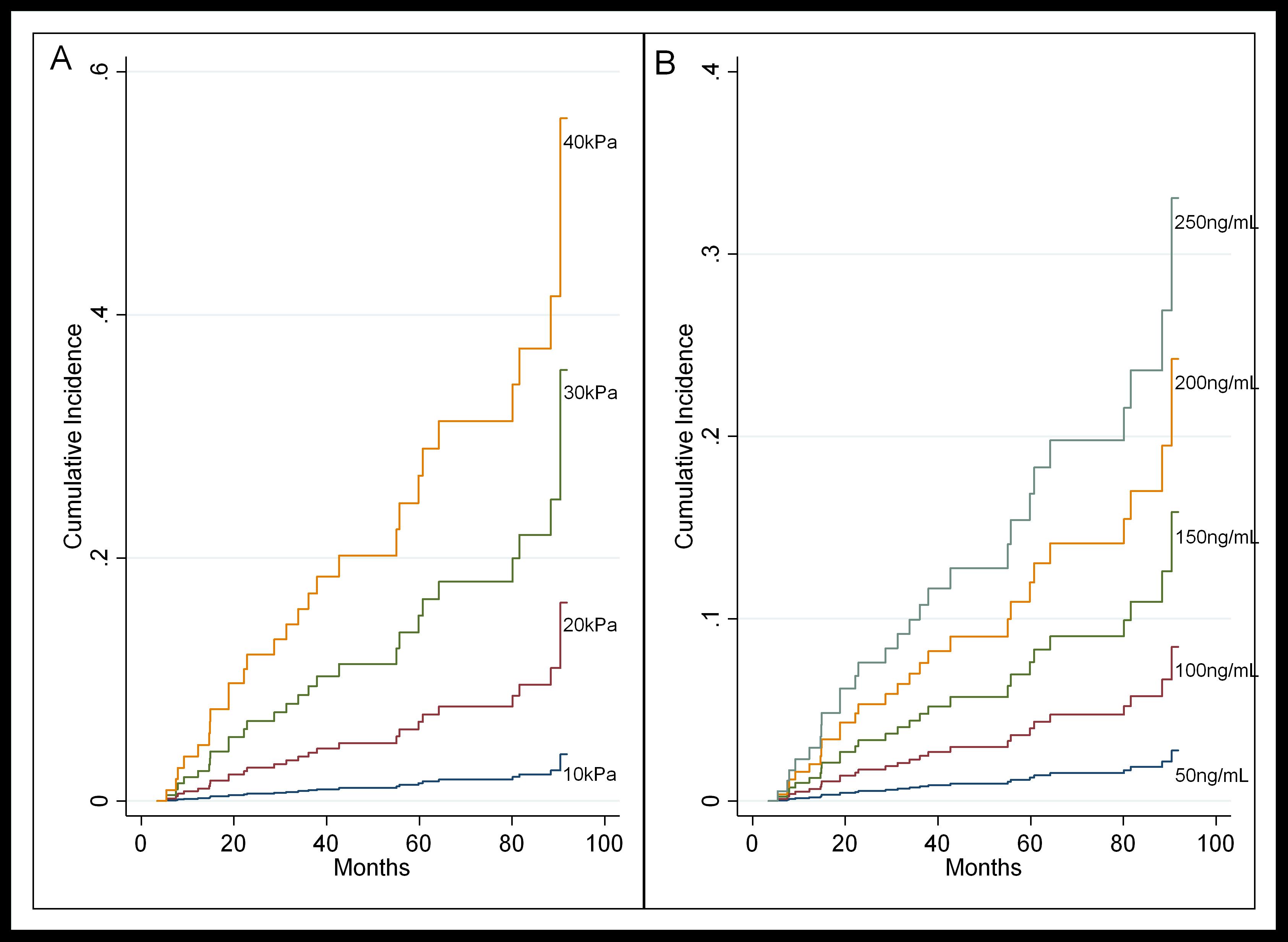

Supplement: S2 Fig — Cumulative incidence for liver related death by different cut-offs for LSM (A) and HA (B). (JPG) [file pone.0212036.s002.jpg]

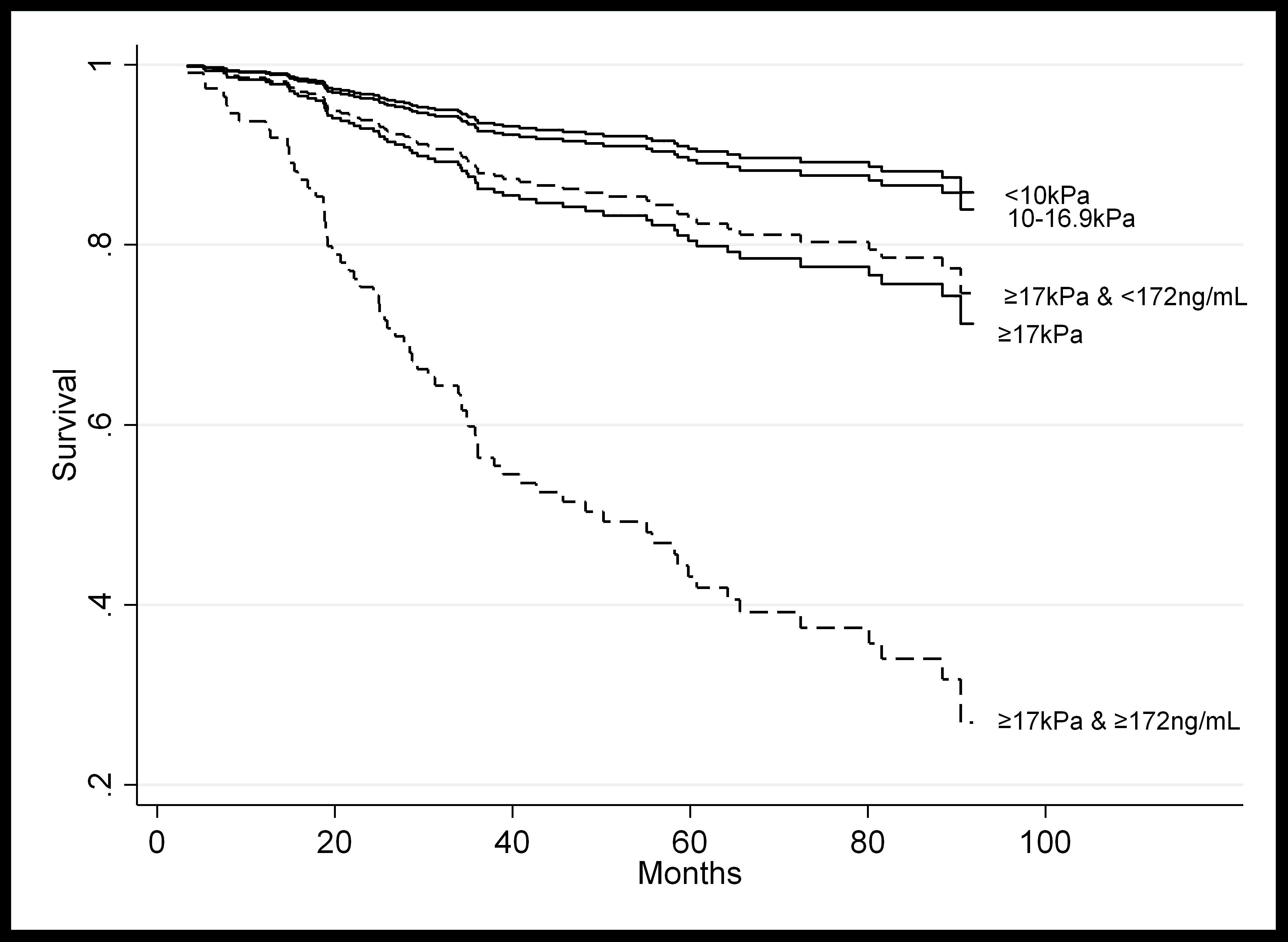

Supplement: S3 Fig — Overall survival by baseline LSM (solid lines) and the LSM 17-75kPa group stratified by the optimized baseline HA cut-off (dotted lines). (JPG) [file pone.0212036.s003.jpg]

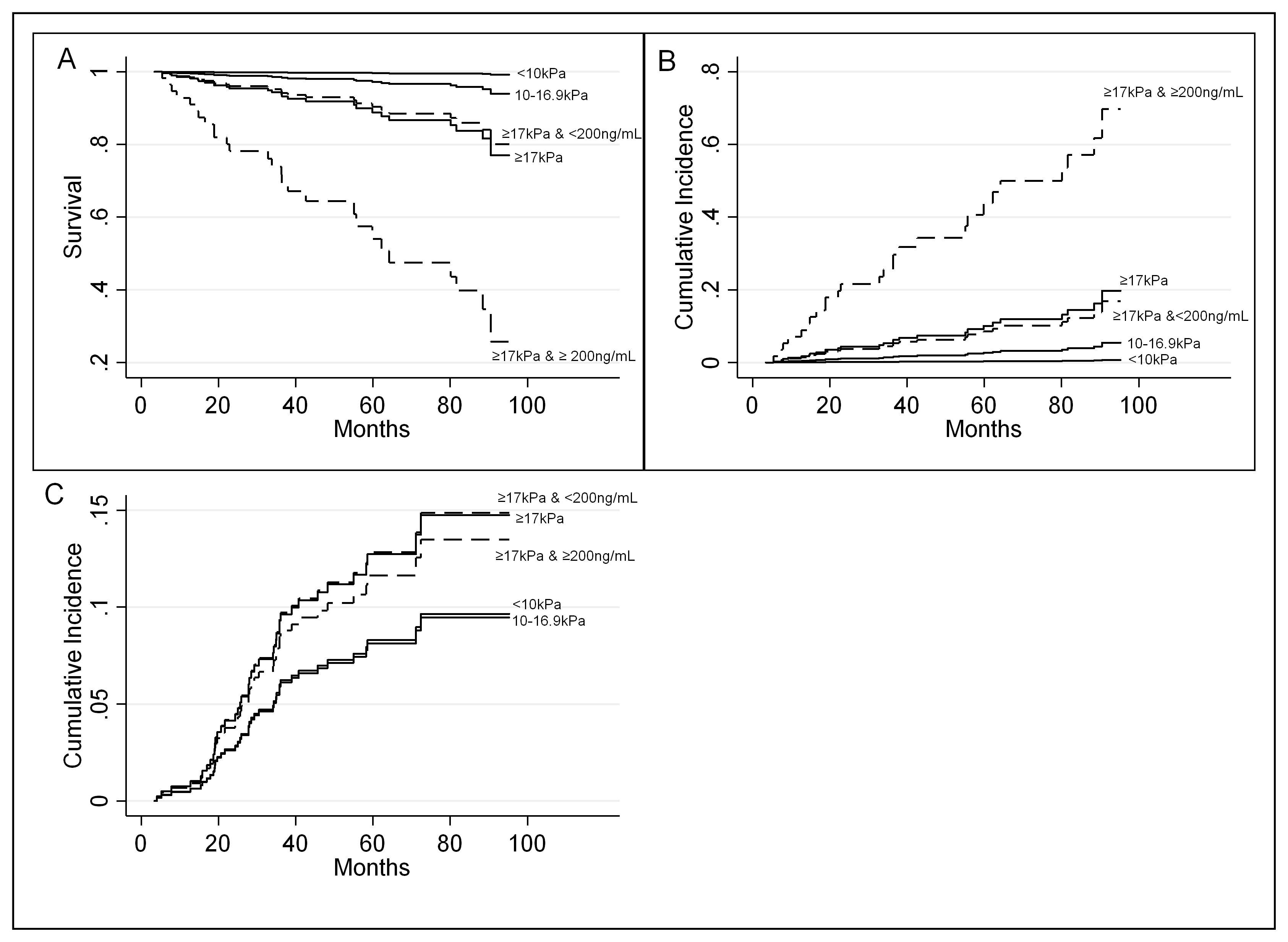

Supplement: S4 Fig — Liver-death free survival (A) by cox regression and (B) cumulative incidence for liver related deaths. (C) Shows the cumulative incidence of death with liver-related deaths as competing risk. All are stratified by LSM (solid lines) and in the LSM 17-75kPa group, the patients are further stratified by baseline HA (dotted lines). (PNG) [file pone.0212036.s004.png]

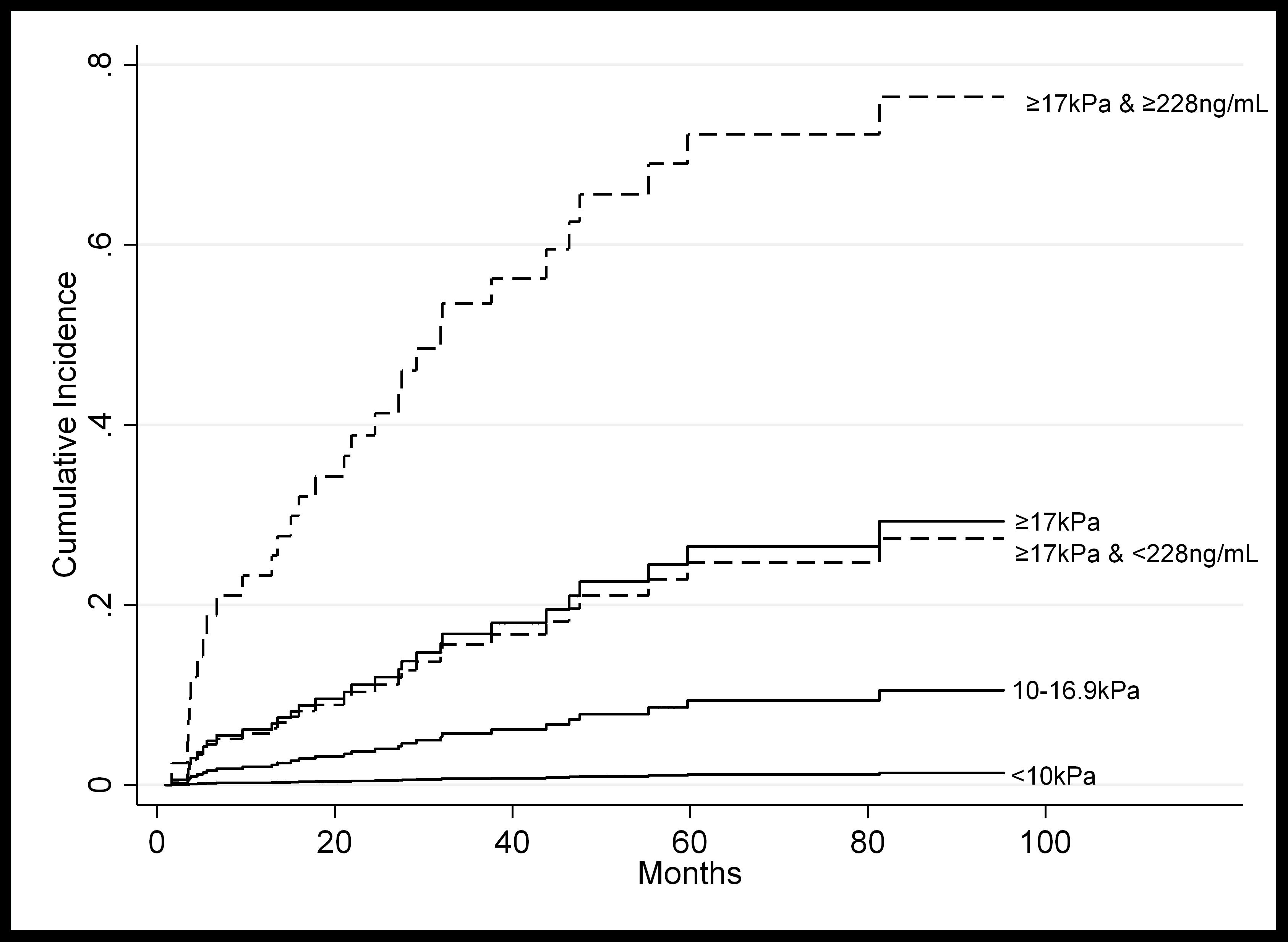

Supplement: S5 Fig — Baseline LSM are the solid lines and the group with baseline LSM 17-75kPa stratified by the optimized cut-off for baseline HA (dotted lines). (JPG) [file pone.0212036.s005.jpg]

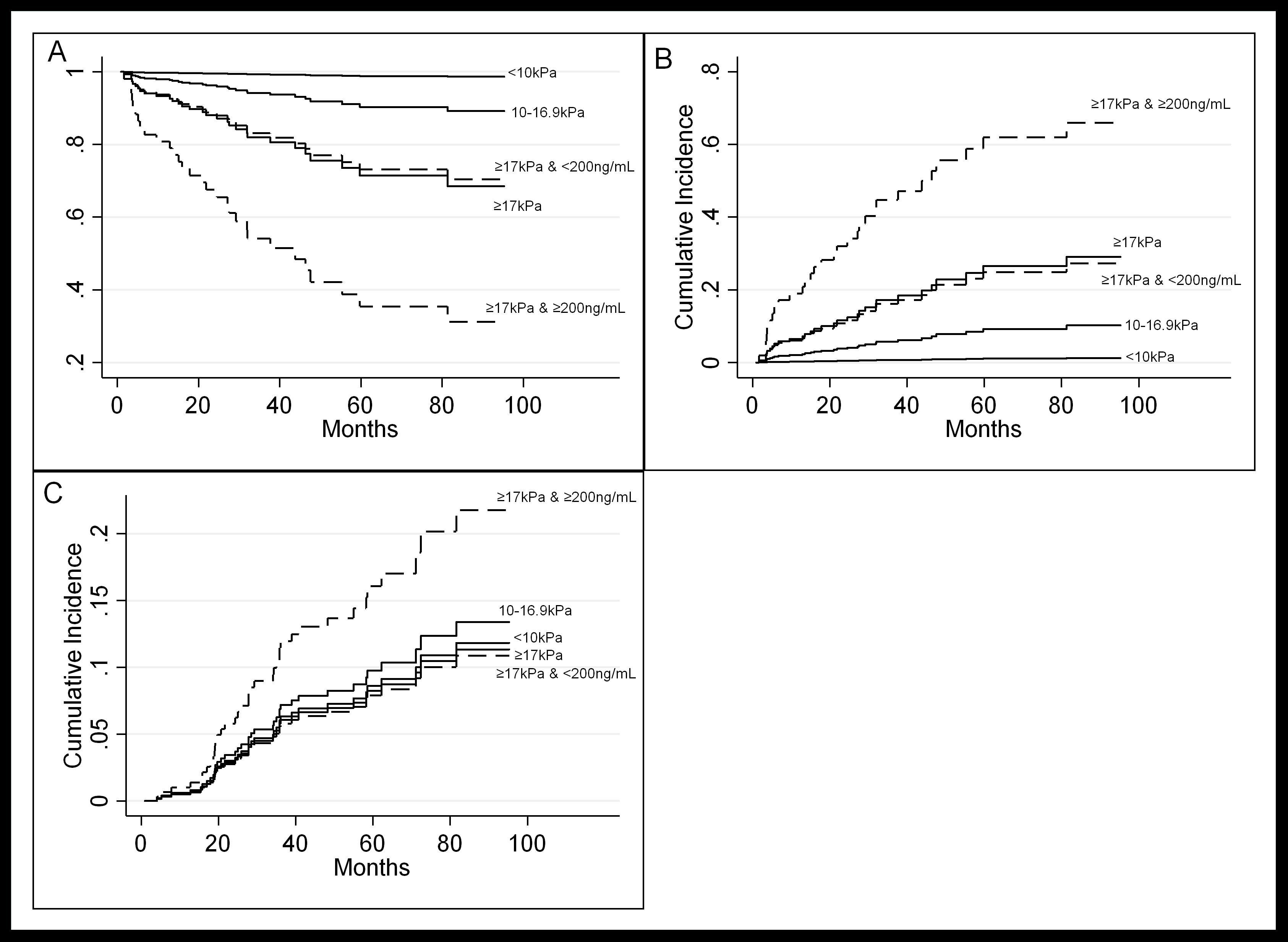

Supplement: S6 Fig — The complication-free survival (A) using cox regression for the event of developing complications to cirrhosis and (B) the cumulative incidence of complications with death as competing risk. (C) shows the cumulative incidence of death with complications as the competing event. All are stratified by LSM (solid lines) and in the LSM 17-75kPa group, the patients are further stratified by baseline HA (dotted lines). (JPG) [file pone.0212036.s006.jpg]

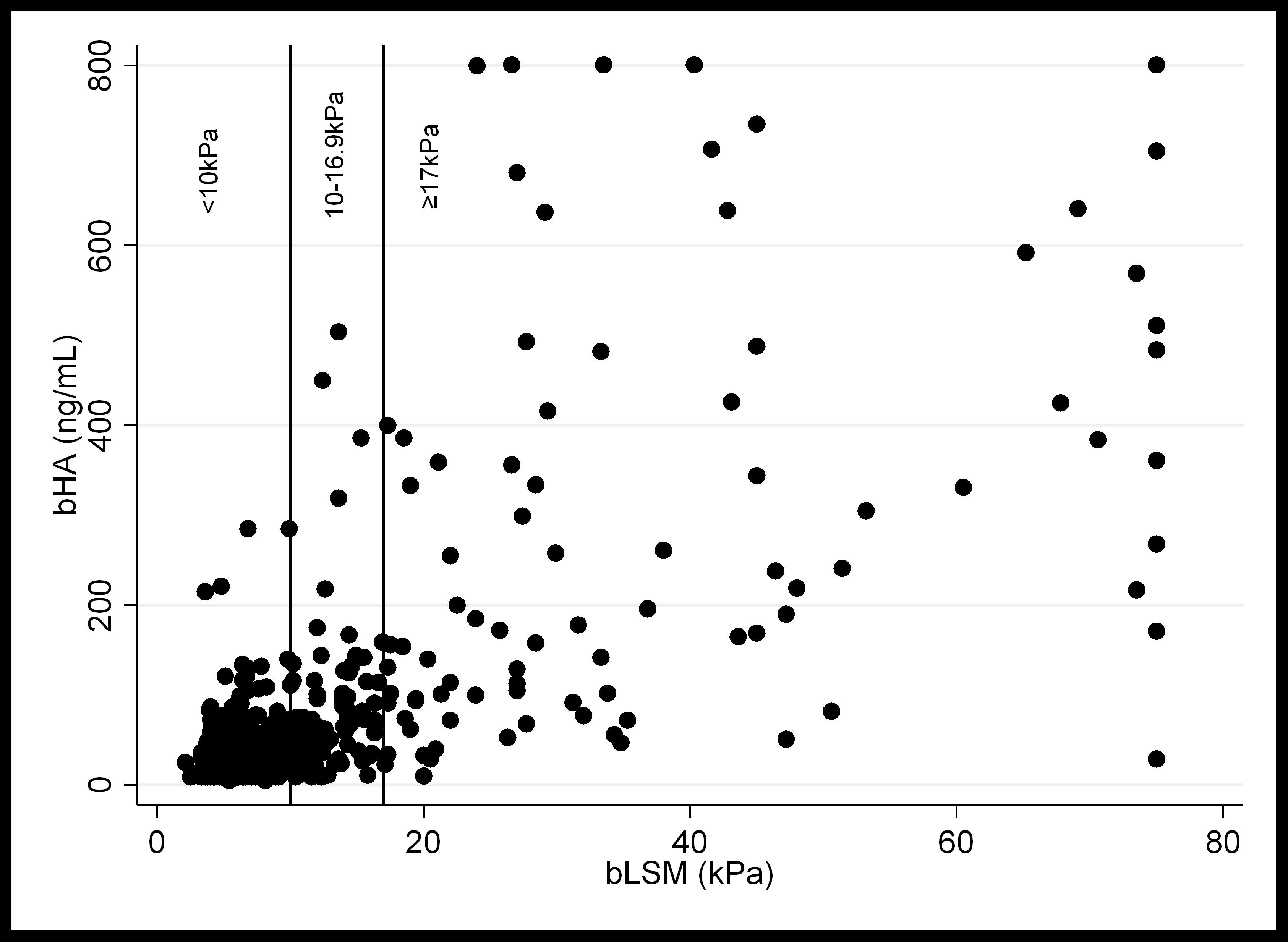

Supplement: S7 Fig — The two vertical lines represents the 10kPa and the 17kPa cut-offs. (JPG) [file pone.0212036.s007.jpg]
