# Supplementary material for: Combining liver stiffness with hyaluronic acid provides superior prognostic performance in chronic hepatitis C
Source: PLoS One. 2019 Feb 11;14(2):e0212036. doi: 10.1371/journal.pone.0212036 (PMC6370278; doi:10.1371/journal.pone.0212036)
Supplement: S1 Table — P-value for difference in median. (DOCX) [file pone.0212036.s008.docx]

| **Median probe (521/591)** | **XL probe (70/591)** | **p-value** |
| --- | --- | --- |
| 6.8kPa (IQR 5.4-75) | 6.9kPa (IQR 5.3-14.6) | 0.86 |
