# Supplementary material for: Combining liver stiffness with hyaluronic acid provides superior prognostic performance in chronic hepatitis C
Source: PLoS One. 2019 Feb 11;14(2):e0212036. doi: 10.1371/journal.pone.0212036 (PMC6370278; doi:10.1371/journal.pone.0212036)
Supplement: S2 Table — The association with LSM and an interaction between probe and LSM was tested and none were significant. Medium probe is the reference. * Subhazard ratios with death from non-liver related causes as competing risk among the +30 years old. (DOCX) [file pone.0212036.s009.docx]

| **Event** | **HR or sHR for XL probe (95% CI)** | **p-value** |
| --- | --- | --- |
| Death | 0.71 (0.26-1.99) | 0.521 |
| Death from liver disease* | 1.18 (0.29-4.76) | 0.814 |
| Complications* | 0.37 (0.07-1.96) | 0.214 |
