# Supplementary material for: Combining liver stiffness with hyaluronic acid provides superior prognostic performance in chronic hepatitis C
Source: PLoS One. 2019 Feb 11;14(2):e0212036. doi: 10.1371/journal.pone.0212036 (PMC6370278; doi:10.1371/journal.pone.0212036)
Supplement: S3 Table — (DOCX) [file pone.0212036.s010.docx]

|  | Baseline variable | Univariate | | Multivariate | |
| --- | --- | --- | --- | --- | --- |
|  |  | **HR (95% CI)** | **p-value** | **HR (95% CI)** | **p-value** |
| Predictors of all-cause mortality | Baseline LSM   - <10kPa - 10-16.9kPa - ≥ 17kPa | Reference  1.35 (0.64-2.79)  5.52 (3.28-9.26) | -  0.43  <0.005 | Reference  1.08 (0.5-2.3)  3.62 (1.64-7.97) | 0.874  0.001 |
|  | Baseline lnHA | 1.82 (1.52-2.19) | <0.005 | 1.36 (1.01-1.84) | 0.041 |
|  | Baseline age | 1.03 (1.01-1.06) | 0.009 | 1.001 (0.98-1.03) | 0.885 |
|  | SVR | 0.46 (0.19-1.15) | 0.096 | 0.32 (0.13-0.79) | 0.014 |
