# Supplementary material for: Combining liver stiffness with hyaluronic acid provides superior prognostic performance in chronic hepatitis C
Source: PLoS One. 2019 Feb 11;14(2):e0212036. doi: 10.1371/journal.pone.0212036 (PMC6370278; doi:10.1371/journal.pone.0212036)
Supplement: S4 Table — Cut-offs for 90% sensitivity and 90% specificity for combined logistic models for all-cause death, liver related death and first cirrhotic complication a n = 560 (no patients <30years of age). b n = 531 (no patients <30years of age or with complications prior to or at inclusion). (DOCX) [file pone.0212036.s011.docx]

|  | | Cut-off | % above cut-off | sens | spe | PPV | NPV |
| --- | --- | --- | --- | --- | --- | --- | --- |
| Combined ln(LSM) and ln(HA) logistic models | **All-cause death** | | | | | | |
|  | Cut-off for 90% sensitivity | 0.0431 | 85.8 | 90.0 | 13.4 | 11.9 | 91.2 |
|  | Cut-off for 90% specificity | 0.1909 | 14.2 | 47.1 | 90.0 | 38.0 | 92.9 |
|  | **Liver-related death^a^** | | | | | | |
|  | Cut-off for 90% sensitivity | 0.0344 | 15.0 | 90.0 | 86.7 | 25 | 99.4 |
|  | Cut-off for 90% specificity | 0.0555 | 14.1 | 88.9 | 90.0 | 30 | 99.5 |
|  | **First cirrhotic complication^b^** | | | | | | |
|  | Cut-off for 90% sensitivity | 0.0421 | 20.3 | 90.0 | 84.8 | 26.0 | 99.3 |
|  | Cut-off for 90% specificity | 0.0757 | 14.7 | 83.3 | 90.0 | 33.1 | 98.9 |
