# Supplementary material for: Combining liver stiffness with hyaluronic acid provides superior prognostic performance in chronic hepatitis C
Source: PLoS One. 2019 Feb 11;14(2):e0212036. doi: 10.1371/journal.pone.0212036 (PMC6370278; doi:10.1371/journal.pone.0212036)
Supplement: S5 Table — Competing risk regression with subhazard ratio for liver related death for all patients (n = 591) including the interaction between age and lnHA. (DOCX) [file pone.0212036.s012.docx]

|  | Baseline variable | Univariate | | Multivariate | |
| --- | --- | --- | --- | --- | --- |
|  |  | **sHR (95% CI)** | **p-value** | **sHR (95% CI)** | **p-value** |
| Predictors of liver related death | Baseline LSM   - <10kPa - 10-16.9kPa - ≥ 17kPa | Reference  10.3 (1.1-98.9)  98.6 (13.5-722.3) | 0.043  <0.0005 | Reference  3.3 (0.34-32.7)  9.5 (1.1-81.1) | 0.232  0.018 |
|  | Baseline lnHA | 3.6 (2.5-5.2) | <0.0005 | 38.7 (6.2-242) | <0.005 |
|  | Baseline age | 1.07 (1.04-1.1) | <0.0005 | 1.31 (1.08-0.63) | 0.006 |
|  | SVR | 1.08 (0.36-3.21) | 0.891 | 0.76 (0.25-2.35) | 0.635 |
|  | Interaction age/lnHA | - |  | 0.95 (0.92-0.98) | 0.004 |
