# Supplementary material for: Combining liver stiffness with hyaluronic acid provides superior prognostic performance in chronic hepatitis C
Source: PLoS One. 2019 Feb 11;14(2):e0212036. doi: 10.1371/journal.pone.0212036 (PMC6370278; doi:10.1371/journal.pone.0212036)
Supplement: S6 Table — Death from liver-related causes is the competing risk. (DOCX) [file pone.0212036.s013.docx]

| Event | Predictors | Univariate | |
| --- | --- | --- | --- |
|  |  | **sHR (95% CI)** | **p-value** |
| NON - Liver related death (n=41) | Baseline LSM   - <10kPa - 10-16.9kPa - ≥ 17kPa | Reference  0.94 (0.41-2.14)  1.42 (0.66-3.07) | 0.883  0.362 |
|  | Baseline lnHA | 0.97 (0.72-1.30) | 0.846 |
|  | Baseline age | 1.001 (0.96-1.31) | 0.887 |
|  | Male sex | 1.35 (0.68-2.66) | 0.383 |
|  | Ever excessive alcohol use | 0.82 (0.44-1.54) | 0.533 |
|  | Ever intravenous drug use | 0.62 (0.27-1.42) | 0.260 |
|  | SVR | 0.15 (0.02-1.08) | 0.06 |
