# Supplementary material for: Combining liver stiffness with hyaluronic acid provides superior prognostic performance in chronic hepatitis C
Source: PLoS One. 2019 Feb 11;14(2):e0212036. doi: 10.1371/journal.pone.0212036 (PMC6370278; doi:10.1371/journal.pone.0212036)
Supplement: S7 Table — Baseline LSM and the time from the last complication to bLSM for the 29 patients with complications prior to or at the time of first reliable LSM and the complications registered. (DOCX) [file pone.0212036.s014.docx]

| First scan median (kPa) | Days since last complication | Variceal bleeding | HCC | Decompensation |
| --- | --- | --- | --- | --- |
| 18.5 | 3452 |  | 1 | 1 |
| 53.2 | 1988 | 1 | 1 |  |
| 28.4 | 1748 | 1 |  | 1 |
| 16.4 | 1528 | 1 |  |  |
| 33.3 | 1195 | 1 |  | 1 |
| 29.3 | 972 | 1 | 1 |  |
| 22 | 957 | 1 |  |  |
| 50.6 | 595 |  |  | 1 |
| 26.6 | 594 | 1 | 1 | 1 |
| 65.2 | 536 | 1 | 1 | 1 |
| 24 | 528 |  | 1 |  |
| 21.3 | 469 |  | 1 | 1 |
| 47.2 | 457 | 1 | 1 |  |
| 75 | 409 |  |  | 1 |
| 27 | 378 |  |  | 1 |
| 38 | 244 | 1 |  | 1 |
| 75 | 209 |  |  | 1 |
| 17.3 | 180 | 1 |  |  |
| 15.7 | 158 | 1 |  |  |
| 41.6 | 115 | 1 |  | 1 |
| 75 | 110 | 1 |  | 1 |
| 75 | 92 | 1 |  | 1 |
| 40.3 | 68 | 1 |  | 1 |
| 69.1 | 64 | 1 | 1 |  |
| 10.1 | 56 |  | 1 |  |
| 45 | 22 | 1 |  | 1 |
| 15.5 | 14 |  | 1 |  |
| 11.9 | 0 | 1 | 1 | 1 |
| 33.3 | 0 | 1 |  |  |
