# Supplementary material for: Combining liver stiffness with hyaluronic acid provides superior prognostic performance in chronic hepatitis C
Source: PLoS One. 2019 Feb 11;14(2):e0212036. doi: 10.1371/journal.pone.0212036 (PMC6370278; doi:10.1371/journal.pone.0212036)
Supplement: S8 Table — * Subhazard ratios with death from non-liver related causes as competing risk among the +30 years old.** subhazard ratio for the patients >30years of age and without prior complications. (DOCX) [file pone.0212036.s015.docx]

| Event | HR or sHR for lnHA (95% CI) | P value |
| --- | --- | --- |
| Death | 1.1 (0.72-1.69) | 0.665 |
| Death from liver disease* | 3.8 (1.54-9.38) | 0.004 |
| Complications** | 2.7 (1.20-5.46) | 0.015 |
