# Supplementary material for: Combining liver stiffness with hyaluronic acid provides superior prognostic performance in chronic hepatitis C
Source: PLoS One. 2019 Feb 11;14(2):e0212036. doi: 10.1371/journal.pone.0212036 (PMC6370278; doi:10.1371/journal.pone.0212036)
Supplement: S9 Table — (DOCX) [file pone.0212036.s016.docx]

|  | HR univariate | p-value | HR multivariate | p-value |
| --- | --- | --- | --- | --- |
| <10kPa  10-16.9kPa  ≥17kPa | Reference  1.31 (0.61-2.8)  6.25 (3.7-10.7) | 0.483  <0.0005 | Reference  1.03  3.56 | 0.942  0.002 |
| lnHA | 1.86 (1.52-2.28) | <0.0005 | 1.3 (0.96-1.75) | 0.091 |
