# Supplementary material for: Combining liver stiffness with hyaluronic acid provides superior prognostic performance in chronic hepatitis C
Source: PLoS One. 2019 Feb 11;14(2):e0212036. doi: 10.1371/journal.pone.0212036 (PMC6370278; doi:10.1371/journal.pone.0212036)
Supplement: S10 Table — Competing risk regression for the non-SVR patients +30years of age (n = 493). (DOCX) [file pone.0212036.s017.docx]

|  | sHR univariate | p-value | sHR multivariate | p-value |
| --- | --- | --- | --- | --- |
| <10kPa  10-16.9kPa  ≥17kPa | Reference  10.31 (1.07-99)  95 (12.8-706) | 0.043  <0.0005 | Reference  4.42 (0.44-44.56)  10.68 (1.10-103) | 0.207  0.041 |
| lnHA | 5.11 (3.28-7.96) | <0.0005 | 2.96 (1.59-5.49) | 0.001 |
