# Supplementary material for: Combining liver stiffness with hyaluronic acid provides superior prognostic performance in chronic hepatitis C
Source: PLoS One. 2019 Feb 11;14(2):e0212036. doi: 10.1371/journal.pone.0212036 (PMC6370278; doi:10.1371/journal.pone.0212036)
Supplement: S11 Table — Patients +30years of age, no previous complications (n = 493). (DOCX) [file pone.0212036.s018.docx]

|  | sHR univariate | p-value | sHR multivariate | p-value |
| --- | --- | --- | --- | --- |
| <10kPa  10-16.9kPa  ≥17kPa | Reference  8.77 (2.22-34.58)  54.64 (16.3-183) | 0.002  <0.0005 | Reference  3.43 (0.9-13)  7.64 (2.1-27.8) | 0.070  0.002 |
| lnHA | 5.02 (3.78-6.65) | <0.0005 | 3.12 (2.22-4.38) | <0.0005 |
