# Supplementary material for: Combining liver stiffness with hyaluronic acid provides superior prognostic performance in chronic hepatitis C
Source: PLoS One. 2019 Feb 11;14(2):e0212036. doi: 10.1371/journal.pone.0212036 (PMC6370278; doi:10.1371/journal.pone.0212036)
Supplement: S12 Table — (DOCX) [file pone.0212036.s019.docx]

| Event | n | Mean months (range) |
| --- | --- | --- |
| Death | 68 | 33.8 (4.1-92.1) |
| Liver related death | 27 | 39.3 (5.4-92.1) |
| Complications | 30 | 23.7 (1.6-81.3) |
