# Supplementary material for: Combining liver stiffness with hyaluronic acid provides superior prognostic performance in chronic hepatitis C
Source: PLoS One. 2019 Feb 11;14(2):e0212036. doi: 10.1371/journal.pone.0212036 (PMC6370278; doi:10.1371/journal.pone.0212036)
Supplement: S13 Table — (DOCX) [file pone.0212036.s020.docx]

|  | Univariate HR | P value | Multivariate HR | p-value |
| --- | --- | --- | --- | --- |
| Baseline LSM   - <10kPa - 10-16.9kPa - ≥ 17kPa | 1  1.34 (0.64-2.78)  5.12 (2.97-8.82) | 0.434  <0.005 | 1  1.001 (0.46-2.16)  2.5 (1.1-5.7) | 0.998  0.028 |
| Ln(HA) | 1.88 (1.52-2.32) | <0.005 | 1.45 (1.07-2) | 0.017 |
