# Supplementary material for: Combining liver stiffness with hyaluronic acid provides superior prognostic performance in chronic hepatitis C
Source: PLoS One. 2019 Feb 11;14(2):e0212036. doi: 10.1371/journal.pone.0212036 (PMC6370278; doi:10.1371/journal.pone.0212036)
Supplement: S14 Table — (DOCX) [file pone.0212036.s021.docx]

|  | Univariate sHR | P value | Multivariate sHR | p-value |
| --- | --- | --- | --- | --- |
| Baseline LSM   - <10kPa - 10-16.9kPa - ≥ 17kPa | 1  9.3(0.96-89.2)  80.4 (10.8-597) | 0.054  <0.0005 | 1  3.3 (0.34-32)  5.9 (0.79-45.3) | 0.301  0.084 |
| Ln(HA) | 5.8 (3.64-9.249 | <0.0005 | 4.02 (2.36-6.82) | <0.005 |
